# Address

**Published:** 1882-06

**Authors:** S. B. Brown

**Affiliations:** Fort Wayne, Indiana


### ADDRESS.

Delivered to the Graduating Class of the Indiana Dental College, at the Third Annual Commencement, February 28, 1882, by Dr. S. B. Brown, D.D.S., of Fort Wayne, Indiana.

Gentlemen of the Graduating Class:—From the moment you first determined to become dentists you have looked forward with great solicitude to this day. The least hopeful have, no doubt, had misgivings that are now set at rest—the crisis has passed, and to you has been the victory. You have been declared by the faculty of the Indiana Dental College to be well worthy of its diploma—no formal, empty honor, I assure you. To them, as well as yourselves, too much credit cannot be given for bringing you to this high standard. This is most emphatically commencement day with you. To-day you have entered upon a new era in your lives. It is a time-honored custom for one of our profession to be invited to offer a few words of advice and encouragement to the class on occasions like this. This honor has fallen upon one who makes no pretensions in the profession outside of the daily routine of an office practice. The suggestions are here offered only with the hope that "what comes from the heart may go to the heart."

In behalf of the College authorities and the dental profession, I cordially extend to you the right hand of fellowship, and congratulate you upon entering a profession that has such a field for progress—a profession in which the highest type of manhood may be attained. In its pursuits there are no temptations to deceive—all the ends are best met by a full statement of the truth. They who are best informed submit with the most confidence to the difficult and painful operations with which we are intrusted. A profession that has been declared "the latest and greatest upheaval of science." With these well-earned honors it is presumed you go forth as doctors of dental surgery to practical life. To succeed, you must enjoy the public confidence. This you can not do unless you deserve it. The community in which you reside will be more observant of your "daily walks and conversation" than you may at first realize. To be a good dentist is not a passport to success. You must be true professional men—gentlemen, honest and pure. Cultivate your minds, as you will be in professional contact with the refined and educated; they will rightly judge from your brain what your handicraft must be. In receiving these diplomas you assume an obligation to protect and elevate your profession. It imposes restraints upon you which you must at all times regard. In your daily practice you must be prepared to meet the peculiarities of each case as it is presented, all to be regarded as special and interesting; it is always of importance to your patient, frequently magnified, but all their views, however erroneous, should be treated considerately by you. Our operations are often attended with much pain; it is important that we extend sympathy to the sufferer, not that of an affected sycophant, but genuine; nothing else will pass; if you lack it, cultivate it that it may be real. Nothing will prepossess your patient more than to feel that you do not willingly inflict pain. Never doubt that they experience all they give evidence of; it is better to act upon this presumption than upon any incredulity—in a word, you must be in harmony with your patient, with sufficient firmness to accomplish the end in view. Let your offices be so arranged that your visitors will recognize refinement and taste, and, above all, so that they may be attractive to yourselves. In the first years of your practice it is not likely you will be fully occupied; it is necessary that you now guard against habits of indolence. I would recommend that you interest yourselves in some study best suited to your tastes collateral with dentistry. Microscopy affords a profitable one, fascinating and instructive, revealing to you the beauties of a world unseen by the unaided vision. Of your personal habits, what shall I say? This is most important of all. It is your character. There is one evil—one enemy to mankind, upon which so many valuable lives are wrecked, that I feel my duty would not be done if I did not warn you against it. It is the use of intoxicants. It is the broad stepping-stone down to degradation, and the first drink is the first step. Let me entreat of you never to "taste, touch or handle" from this hour. In the curriculum of this college I believe Watt's chemical essays are included. This volume contains language in extolling nature's beverage that may possibly have failed to impress you. It is so pertinent to my advice to you on this point, that I quote it:—

"In the day that God created these wonderful elements, positive and negative, created He them, and blessed them, and called their name water in the day when they were created. The drink that a God of love has distilled for all of his children. The liquor that strengthens, refreshes, revives, invigorates and purifies. The beverage which has no orphan's tear as its sequel, nor widow's wail as a requiem over its fallen victims, a drink that demons do not delight in. The murderer does not imbibe it to prepare him for his crime, nor the reveler as a prelude to his midnight debauch. No ghosts of murdered innocents awake from their slumbers to curse the cup that contains it, or the fountain that gushes it forth. Dethroned reason does not curse and babble under its influence, nor does delirium travel in its wake. The lone prisoner does not accuse it of the crime that has brought him to the dungeon cell; nor does the felon on the scaffold curse the "moss covered bucket" for his untimely end. Prisons and alms houses do not overflow with its victims; nor are courts of justice kept busy with its crimes."

In your business intercourse be always known as reliable. Let your word be as good as your bond. Never go in debt beyond your ability to meet at a stated time. Never let a creditor call on you but once; after that make the calls yourself. Let it never be said of you that you made a promise which you did not redeem. The most odious members of any community are those who do not pay their debts. Thus, by being prompt, you can consistently exact promptness in return for your services. Now, having urged upon you this consideration for others, it is but justice to yourselves and indispensable to your success, that you require adequate compensation for what you render to others. Dentistry is a liberal profession, and you should be ready and willing to treat those who are unable to offer a return without a desire for reward; but for those not having such claims, never fail to fully estimate your own labor, if you would have others do likewise. You cannot progress as dentists nor become good citizens without means to discharge your pecuniary obligations, and it is your fault if your profession does not, like other learned and liberal professions, command it. Now, in parting, let me assure you that this is not an occasion for the severance of ties that have been formed and bind you to your faculty, but on the contrary they are now strengtened by a brotherhood. They who have labored with you so long and unselfishly will always esteem it a pleasure to encourage and counsel you as you go forward in the battle of life. I am sure, with their noble example before you, you will be an honor to your Alma Mater and a credit to yourselves. Farewell, and God bless you.

